# Raw dataset of tensile tests in a 3D-printed nylon reinforced with oriented short carbon fibers

**DOI:** 10.1016/j.dib.2024.111149

**Published:** 2024-11-20

**Authors:** Ênio H. Pires, João V. Barreto Netto, Marcelo L. Ribeiro

**Affiliations:** Aeronautical Engineering Department, São Carlos School of Engineering. *Av*. João Dagnone, 1100, São Carlos, SP 13563-120, Brazil

**Keywords:** Tensile test, 3D-printing, Composite materials, Nylon, Onyx, Digital image correlation

## Abstract

This dataset presents the results of tensile tests conducted on 3D-printed nylon composites reinforced with short carbon fibers, commercially known as *Onyx™*. Specimens were printed using a *Markforged™* Mark 2 printer with three different printing orientations: 0°, ±45°, and 90°, following the ASTM D638-22 standard for Type IV tensile specimens. The dataset includes mechanical testing data, scanning electron microscope (SEM) images, and digital image correlation (DIC) images. Mechanical test data were collected using an Instron universal testing machine, while SEM images were captured to examine microstructural features and fracture surfaces, both before and after testing. DIC images were obtained using two cameras positioned orthogonally to capture deformation on multiple planes. Limitations include fracture at the radius of the testing region in some 0° specimens and premature failure of 90° specimens, which reduced the number of captured images. These data provide valuable insights into the anisotropic mechanical behavior of 3D-printed composites and can be reused for further research on material behavior under varying conditions like multiscale simulations and deep learning algorithms.

Specifications Table

**Table:** 

Subject	Material Characterization.
Specific subject area	Characterization of a 3D-printing composite material for numerical applications in solid mechanics and structures.
Type of data	Tables and Images*.*
Data collection	The tests were conducted using an Instron universal testing machine equipped with a 250 kN load cell. Two cameras were positioned to capture images on orthogonal planes, capturing pictures every 5 seconds. Scanning Electron Microscope (SEM) images were taken from both undeformed areas and the fracture area after testing, at magnifications of 100×, 500×, and 1000×.
Data source location	Country: Brazil. State: São Paulo. City: São Carlos. Location: Aeronautical Engineering Department. Latitude: −22.0154, Longitude: −47.8911.
Data accessibility	Repository name: Mendeley Data Data identification number: 10.17632/9nnf4vmg8p.2 Direct URL to data: https://data.mendeley.com/datasets/9nnf4vmg8p/2
Related research article	None.

## 1 Value of the Data

• Relevance to Material Science Research: The dataset provides raw tensile test data of a 3D-printed nylon composite reinforced with short carbon fibers printed in 3 different directions, this kind of material and manufacturing process has been increasingly used in advanced engineering applications due to its lightweight and high-strength properties. Researchers studying the mechanical behavior of similar composites will find this dataset valuable for benchmarking and validating their own models.
• Enables Comparison Across Studies: The inclusion of Digital Image Correlation (DIC) data from two orthogonal planes allows for a detailed analysis of Poisson's ratios (*v*_12_ and *v*_13_). This data can be reused by researchers to compare mechanical properties across different composite materials, manufacturing methods, or experimental setups.
• Supports Further Material Characterization: The SEM images provide detailed insights into both undeformed areas and fracture surfaces at various magnifications (100×, 500×, and 1000×). This information can help researchers understand the failure mechanisms and microstructural behavior under tensile loading, facilitating the development of improved composite materials.
• Facilitates Data-Driven Model Development: The comprehensive dataset, including both raw tensile test data and high-resolution images, is ideal for developing and training data-driven models to predict mechanical behavior under various loading conditions. The dataset can serve as a valuable resource for researchers working on machine learning or data-driven approaches in material science.
• Open Access for Extended Studies: The open availability of this dataset enables researchers from various fields, such as mechanical engineering, materials science, and polymer science, to perform meta-analyses, derive new hypotheses, or use the data for educational purposes, ultimately fostering collaboration and advancing knowledge in composite materials.

## 2 Background

Additive manufacturing (AM), or 3D printing, is rapidly advancing, enabling the production of more complex parts. This advancement has expanded its use as a production technique for end-use products in various industries. With the emergence of new techniques, it is now possible to print high-performance materials, such as composites. Additive manufacturing encompasses a wide range of techniques, such as fused filament fabrication (FFF), where these techniques have the capability of producing composite parts with continuum or short fiber to the polymer matrix [1,2].

To fully leverage these 3D printing methods, understanding the material properties and the dependence on process parameters is essential to optimize performance. This dataset can support future research related to the characterization of the mechanical properties of nylon reinforced with oriented short carbon fibers and the application in multi-scale simulations using representative volume elements (RVE) [3]. Also, the raw dataset can be used to feed neural networks to provide novel techniques to predict mechanical behavior of materials, both by regression and/or image modelling [4].

## 3 Data Description

**Fig. 1:**
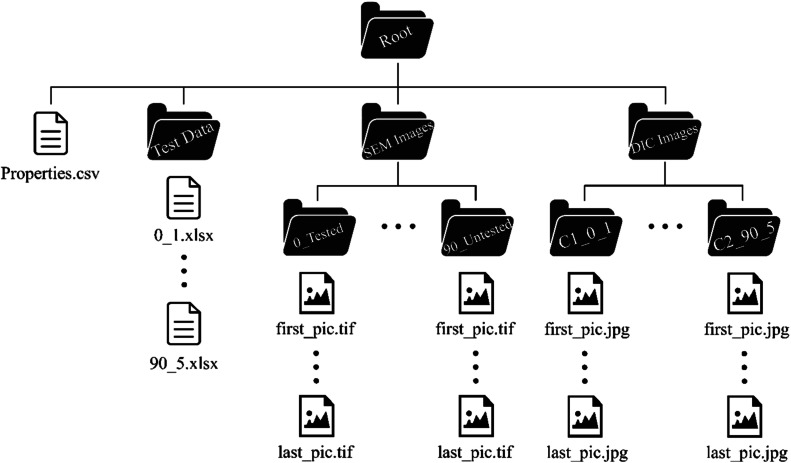
Folder structure of the dataset.

The dataset is organized into three main folders, each named according to the type of data it contains. Additionally, a table in the root directory provides the physical properties of the samples, such as mass and dimensions. The folder structure is illustrated in Fig. 1.

The *'Properties.csv'* file contains 8 columns and 16 rows, with the first row listing the column headers. The first column identifies each specimen, labeled from 0_1 to 0_5 for the 5 specimens printed in the 0° orientation. The same labeling system is applied for the specimens manufactured at the ±45° and 90° orientations, resulting in a total of 15 samples. The second column provides the mass of each specimen in *grams*. The remaining 6 columns present the dimensions of the specimens in *millimeters*, measured along their testing lengths.

The *'Test Data'* folder contains data acquired from the Universal Testing Machine (UTM) in *.xlsx* format. Each file in this folder is named according to the first column in the *'Properties.csv'* file, allowing for easy correlation between the files. Each test data file contains 4 columns and a variable number of rows, depending on the duration of the test. The first row lists the column headers. The first column is a row index, the second column represents the test time in *seconds*, the third column records the displacement of the UTM crosshead in *millimeters*, and the fourth column contains the load data in *Newtons*.

The *'SEM Images'* folder is organized into 6 subfolders, representing images for all three printing configurations, with two subfolders for each configuration. The subfolders are named according to the printing orientation and whether the images are from the tested part of the specimen (fracture region) or the untested part. Consequently, the subfolders are labeled as 0_Tested, 0_Untested, 45_Tested, up to 90_Untested. The images in each subfolder are in *.tif* format, and the number of images varies across subfolders.

The *'DIC Images'* folder is divided into 30 subfolders, corresponding to the specimens tested. Since two cameras were used for each test, the number of subfolders is twice the number of specimens. The subfolder names match those in the *'Test Data'* and *'Properties.csv'* descriptions, with the prefixes 'C1′ and 'C2′ used to distinguish data collected by Camera 1 and Camera 2. Therefore, subfolder names range from C1_0_1, C1_0_2, up to C2_90_4, C2_90_5. The image files within each subfolder are named randomly, but the last number in each file name increases sequentially according to the order in which the images were captured. All DIC image files are in *.jpg* format.

## 4 Experimental Design, Materials and Methods

The dataset was generated from tensile tests performed on 3D-printed nylon specimens reinforced with short carbon fibers. The specimens were manufactured with layer orientations of 0°, ±45°, and 90° to evaluate the anisotropic mechanical properties of the material.

### 4.1 Manufacturing process

The specimens were fabricated using a Mark 2 3D-printer from *Markforged^TM^* by a Fused Filament Fabrication (FFF) method. The fused material was a nylon reinforced with oriented short carbon fibers, commercially known as *Onyx^TM^*. The printing parameters were: 100-micron (0.1 mm) resolution (Mark2 standard), nozzle temperature of approximately 275 °C, and infill density of 100 % (solid infill) [5].

**Fig. 2:**
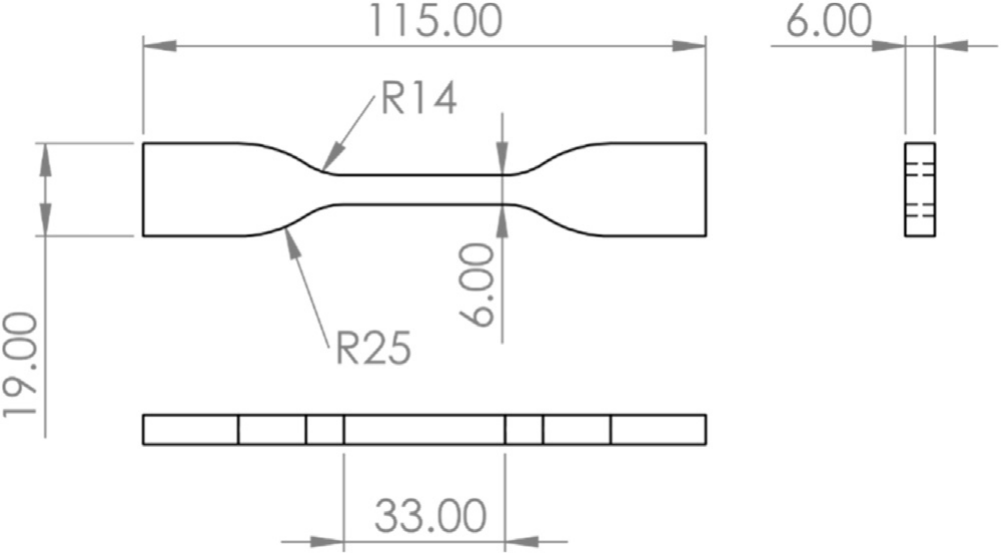
Dimensions and shape of the specimens.

According to the ASTM D638–22 [6], a total of 15 specimens were produced: 5 with 0°, 5 with ±45°, and 5 with 90° printing orientations. The specimen dimensions were based on the Type IV standard. To properly utilize the two cameras for Digital Image Correlation (DIC), the thickness of the specimens was modified from the 3.2 mm specified in the standard to 6.0 mm, matching the width of the testing region, thus featuring a square-shaped cross-sectional area. The dimensions used in the *Eiger* software to generate the slices for printing are shown in Fig. 2.

**Fig. 3:**
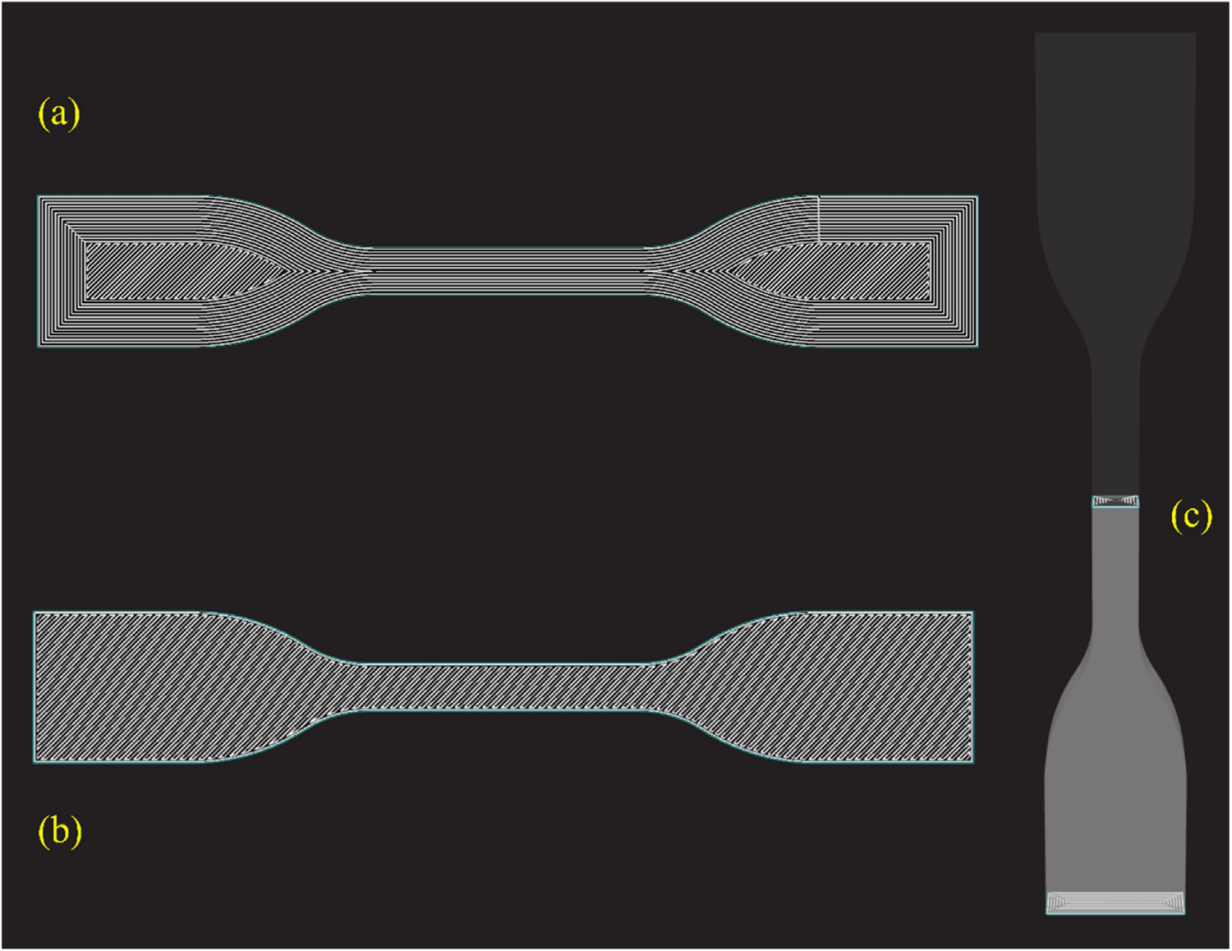
Slicing scheme done on Eiger for (a) 0°, (b) ±45°, and (c) 90°.

The slices were generated in three different configurations. The first configuration was designed to produce specimens printed along the tensile load direction, with a 0° printing orientation. To achieve this, 15 concentric wall layers were selected, ensuring that when the nozzle passed through the testing region, the printing direction was aligned with the load direction. The second configuration was created for specimens with ±45° printing orientations. In this case, only 1 wall layer was used (the minimum allowed by the *Eiger* software), and the rest of the area was sliced according to the standard software orientation. The 0° and ±45° specimens were both flat printed (build orientation). While the third configuration (developed for specimens with a printing orientation perpendicular to the load) were rotated 90° along the x-axis and printed in a vertical position. The slicing scheme is shown in Fig. 3.

**Fig. 4:**
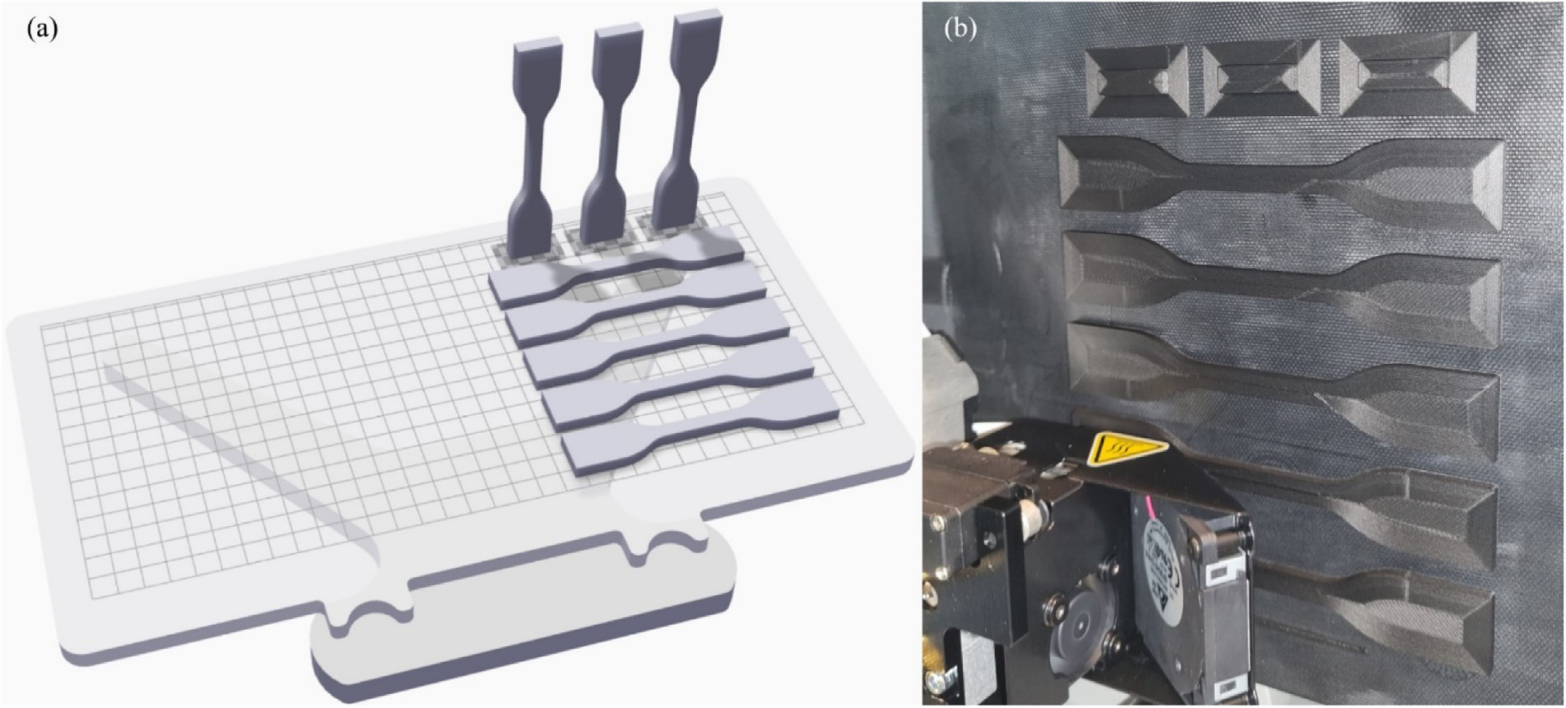
(a) specimens positioned on Eiger and (b) specimens being printed.

The specimens printed with a 90° orientation had a brim at the bottom to support them during printing and prevent them from falling during the process. The specimens were not printed all at once. First, 5 specimens with a 0° orientation were printed alongside 3 specimens with a 90° orientation. Then, 5 specimens with a ±45° orientation were printed, along with the remaining 2 specimens at 90° The layout of the specimens on the printing table, as configured in both the Eiger software and the printer, is shown in Fig. 4.

**Fig. 5:**
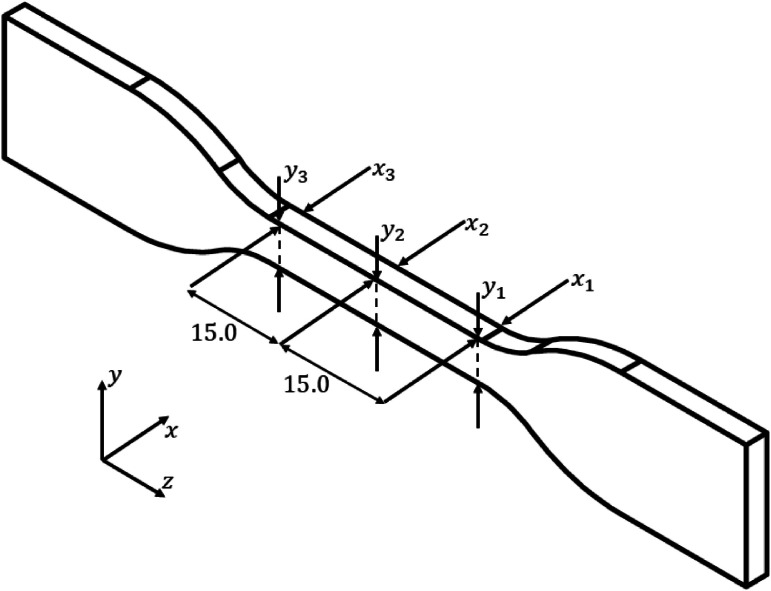
(a) Measurement positions for the specimens.

After the fabrication of the specimens, measurements were taken in three different regions, positions 1, 2, and 3, where dimensions were recorded in the x and y directions, as shown in Fig. 5. A Starrett™ caliper model 125MEB with a resolution of 0.05±0.02 mm was used for these measurements. Subsequently, mass verification was conducted using an OHAUS™ balance model AR3130 with a resolution of 0.001±0.002 g. The measurement data for the dimensions are available in the 'Properties.csv' file. In this file, the first column lists the specimen number, the second column records its mass, and the subsequent columns detail the dimensions at positions x1, x2, x3, y1, y2, and y3.

### 4.2 Experimental setup

The tensile tests were carried out on an Instron universal testing machine by Instron, model 5985, equipped with a 250 kN load cell. The specimens were mounted using mechanical wedge grips to ensure secure clamping and uniform load distribution. The alignment of the specimens was controlled by an alignment device to minimize bending moments during testing. The tests were conducted at controlled temperature of 20 °C.

**Fig. 6:**
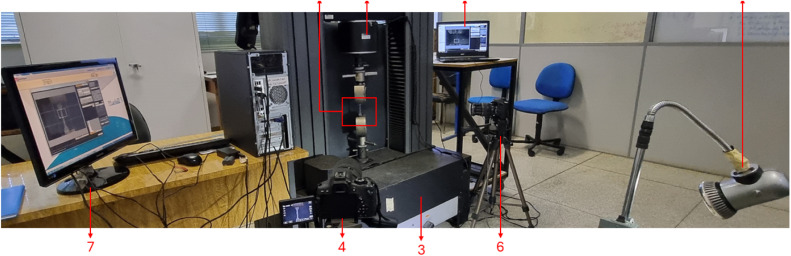
Experimental setup where item 1 is the specimen, 2 is the INSTRON 5985, 3 is the camera 1, 4 is the Data Acquisition Device for Camera 1, 5 is camera 2, 6 is the Data Acquisition Device for Camera 2 and universal testing machine, and 7 is the Lighting Device.

Two high-resolution cameras (Canon EOS Rebel T6i and Canon EOS 350D) were used for Digital Image Correlation (DIC) to capture images of the specimen surfaces during testing. The cameras were positioned orthogonally to one another, with Camera 1 focused on the frontal direction and Camera 2 on the longitudinal direction. The distance between the cameras and the specimens was 1400 mm and 700 for Camera 1 and 2, respectively. Images were captured at intervals of 5 s throughout the test duration to record the progressive deformation of the specimens. The experimental setup is shown in Fig. 6.

The tensile tests were performed following the ASTM D638–22 standard procedure for testing polymer composites. Each specimen was loaded at a constant crosshead speed of 5 mm/min until failure. The load and displacement data were recorded continuously at a sampling rate of 0.1 data/s using the UTM's integrated data acquisition system.

### 4.3 Data Acquisition for DIC

**Fig. 7:**
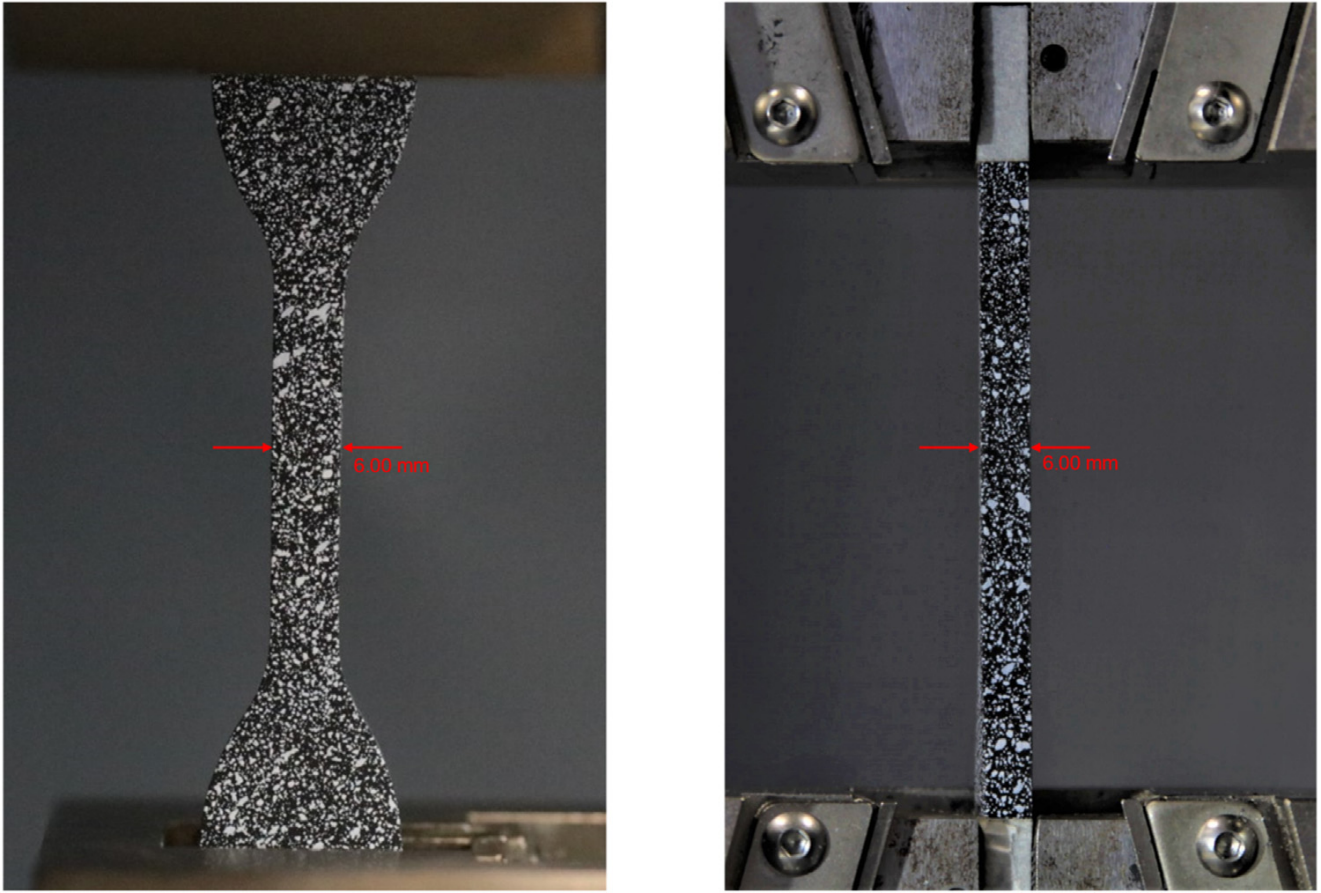
(a) Frontal direction and (b) transverse direction.

The DIC images were acquired simultaneously from two cameras, capturing the strain distribution across different planes of the specimen. Camera 1 (Canon EOS Rebel T6i) was positioned for a frontal view, capturing images at a resolution of 6000×4000 pixels with a focal length of 180 mm, an f-number of f/3.5, an ISO speed of 6400, and an exposure time of 1/125 seconds. Camera 2 (Canon EOS 50D) provided a lateral view, capturing images at a resolution of 4752×3168 pixels with a focal length of 135 mm, and f-number of f/5.6, an ISO speed of 100, and an exposure time of 1/2 s. Proper lighting was used to ensure uniform illumination across the specimen surface, reducing image noise and improving DIC accuracy. The cameras were calibrated using a stereo calibration grid to ensure accurate deformation measurements. Additionally, for the use of DIC, the black specimens were painted with white spray paint, aiming to maintain a standard granularity of 1±0.5 mm. The granularity can be observed in Fig. 7.

### 4.4 SEM imaging procedure

**Fig. 8:**
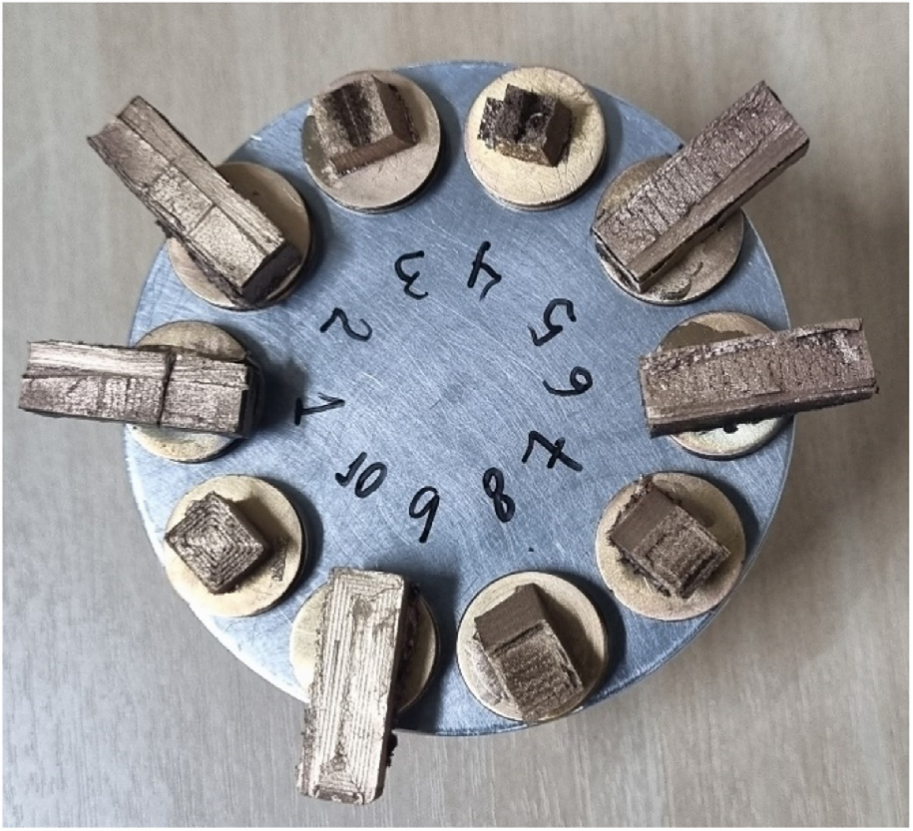
All the SEM specimens after coating.

To analyze the microstructure of the composite before and after testing, the SEM specimens were divided into tested and untested groups. For the tested group, specimens with flatter fracture surfaces were selected to ensure better focus in the microscope images. Two different specimens were analyzed for the 0° and ±45° printing orientations. Since all the 90°-oriented specimens exhibited similar fracture patterns, only one specimen was analyzed by SEM and reported. The same number of samples for each configuration was used for the untested specimens, totalizing 10 different samples that are shown in Fig. 8. The untested SEM specimens were taken from the grip section of the Type IV tensile specimens, as this area remains unaffected by the deformation during the tensile test. To avoid plastic deformation during sectioning, a pre-crack was introduced, and the specimens were submerged in liquid nitrogen for 5 minutes. Subsequently, a brittle fracture by impact was used to expose the undeformed inner part of the material.

The SEM imaging was performed to analyze the microstructural features and fracture surfaces of the specimens. The SEM images were captured using a LEO/ZEISS 440 microscope at magnifications of 100×, 500×, and 1000×. Specimens were sectioned to expose both undeformed regions and fracture surfaces. Prior to imaging, the samples were sputter-coated with a thin layer of gold to prevent charging under the electron beam. The imaging was carried out using a secondary electron detector (SE1) under an accelerating voltage of 15 kV, with a working distance between 18 mm and 20 mm to optimize image quality and resolution.

## Limitations

One limitation observed during testing was the fracture location in four out of the five specimens manufactured with a 0° printing orientation. The fracture occurred at the transition radius where the testing area begins, rather than within the designated gauge length.

Another limitation was related to the testing duration for the specimens printed at a 90° orientation. These specimens fractured prematurely, resulting in a limited number of images being captured during the test. While reducing the test speed could have provided more images for analysis, this adjustment would likely introduce inaccuracies due to the viscoelastic behavior of the polymer, affecting the validity of the mechanical property measurements [7].

## Ethics Statement

The authors have read and follow the for publication in Data in Brief and confirmed that the current work does not involve human subjects, animal experiments, or any data collected from social media platforms.

## CRediT authorship contribution statement

**Ênio H. Pires:** Conceptualization, Methodology, Investigation, Data curation, Writing – original draft. **João V. Barreto Netto:** Conceptualization, Methodology, Investigation, Data curation, Writing – original draft. **Marcelo L. Ribeiro:** Supervision, Resources, Conceptualization, Writing – review & editing.

## Data Availability

Mendeley DataRaw Dataset from Tensile Tests of 3D-Printed Nylon Reinforced with Short Carbon Fibers (Original data).
